# Cytotoxic CD4^+^ T cells in cancer: Expanding the immune effector toolbox

**DOI:** 10.1016/j.immuni.2021.11.015

**Published:** 2021-12-14

**Authors:** David Y. Oh, Lawrence Fong

**Affiliations:** 1 Division of Hematology/Oncology, Department of Medicine, University of California, San Francisco, San Francisco, CA, USA

## Abstract

Cytotoxic T cells are important effectors of anti-tumor immunity. While tumor killing is ascribed to CD8^+^ T cell function, pre-clinical and clinical studies have identified intra-tumoral CD4^+^ T cells that possess cytotoxic programs and can directly kill cancer cells. Cytotoxic CD4^+^ T cells are found in other disease settings including infection and autoimmunity. Here, we review the phenotypic and functional characteristics of cytotoxic CD4^+^ T cells in non-cancer and cancer contexts. We conduct a comparative examination of cytolytic mechanisms of cytotoxic CD4^+^ T cells across disease states and synthesize features that define these cells independent of context. We discuss regulatory mechanisms driving ontogeny and effector function and evidence for the clinical relevance of cytotoxic CD4^+^ T cells in cancer. In this context, we highlight important gaps in understanding in the biology of cytotoxic CD4^+^ T cells as well as the potential use of these cells in immunotherapies for specific cancers.

## INTRODUCTION

Effector functions within the T cell compartment are critical for shaping immune responses in numerous disease states. CD4^+^ T cell effector function is thought to be centered around cytokine production, whereas direct cytotoxic activity against target cells resides within the CD8^+^ T cell compartment. However, these boundaries can be plastic, and studies in pre-clinical and clinical contexts have identified CD4^+^ T cells that not only express key molecules associated with cytolytic granules such as granzymes (GZM) and perforin (PRF1), but also possess direct cytotoxicity that underlies both pathogenic and protective immunity, including in cancer.

Much of the current understanding on the functionality of cytotoxic CD4^+^ T cells comes from studies on anti-viral immunity (reviewed in Cheroutre and Husain, 2013; Marshall and Swain, 2011; Takeuchi and Saito, 2017). Cytotoxic CD4^+^ T are consistently identified in a number of chronic viremic states, including mice infected with lymphocytic choriomeningitis virus and gamma-herpes virus, and patients with human immunodeficiency virus, cytomegalovirus, Epstein-Barr Virus, and hepatitis B and C (Jellison et al., 2005; Soghoian et al., 2012; van Leeuwen et al., 2004; Sáez-Borderías et al., 2006; Suni et al., 2001, Aslan et al., 2006); however, these cells are also generated in acute viremias such as influenza (Brown et al., 2012). In regard to their activation requirements, it is clear that in viremic states, cytotoxic CD4+ T cells recognize cognate viral antigens in a major histocompatibility complex class II (MHC class II)-restricted manner, which mirrors the MHC restriction of non-cytotoxic CD4^+^ T cells (Jellison et al., 2005; Soghoian et al., 2012; Khanna et al., 1997; Adhikary et al., 2006; Landais et al., 2004; Long et al., 2005). This was elegantly demonstrated by single-cell RNA sequencing (scRNA-seq) and T cell receptor sequencing (TCR-seq) of dengue-infected donors where cytotoxic CD4^+^ T cells exhibited an CD45RA+ effector memory phenotype and were enriched for clonotypes that recognized dengue viral antigen in previously infected donors (Patil et al., 2018). The recognition of virally derived peptides by cytotoxic CD4^+^ T cells can lead to target cell killing (Jellison et al., 2005; Soghoian et al., 2012; Brown et al., 2012). Beyond professional antigen-presenting cells, cytotoxic CD4+ T cells can also target other infected cells such as epithelial cells and B cells, which express MHC class II in humans (Hegde et al., 2005; Jellison et al., 2005; van Leeuwen et al., 2006). While there are still enduring questions about the relative contribution of cytotoxic CD4^+^ versus CD8^+^ T cells to viral control, their study in viremic states implicates cytotoxic CD4^+^ T cells as effectors with direct cytolytic activity for target cells.

Cytotoxic CD4^+^ T cells are implicated in disease states outside of viral infection. Notably, in murine models, CD4^+^ CD8α^+^ cells expressing GZM, PRF1, interferon (IFN)-γ, and separately cytotoxic CD4^+^ T cells expressing the class I-restricted T cell-associated molecule (CRTAM), locate within the intraepithelial lymphocyte (IEL) compartment of the intestine (Takeuchi et al., 2016; Mucida et al., 2013; Reis et al., 2013; Reis et al., 2014). To some extent, the generation of these cells may be indirectly protective against autoimmune colitis in a T cell transfer model by diverting CD4^+^ cells toward a quiescent cytotoxic CD4^+^ phenotype and away from a pathogenic Th17 phenotype (Reis et al., 2013); however, when re-challenged with cognate antigen in the context of inflammatory stimuli, cytotoxic CD4^+^ IELs can also exacerbate autoimmune colitis (Mucida et al., 2013). Human autoimmune conditions are also associated with cytotoxic CD4^+^ T cells. In patients with systemic lupus erythematosus (SLE) and B6.MRL/*lpr* mice, NKG2D+ CD4^+^ T cells that express granzyme B (GZMB) and PRF1 can kill regulatory T (Treg) cells in a manner that depends on NKG2D engagement by NKG2D ligand on Treg cells as well as a partial dependence on Fas-Fas ligand engagement (Yang et al., 2017). In addition, in immunoglobulin G4 (IgG4)-related disease, the infiltration of affected organs by cytotoxic CD4^+^ T cells is reduced by glucocorticoids; furthermore, dominant T cell receptor (TCR) β clonotypes in the circulation are shared with tissue-infiltrating cytotoxic CD4^+^ T cells (Della-Torre et al., 2018). Inflamed skin lesions from systemic sclerosis patients are also infiltrated by GZMA+ CD4^+^ T cells, which can be seen directly encountering human leukocyte antigen (HLA)-DR+ endothelial cells undergoing apoptosis, suggesting a direct cytotoxic role in end-organ tissue damage (Maehara et al., 2020). Cytotoxic CD4^+^ T cells are also seen with aging. Increased proportions of clonally expanded, circulating cytotoxic CD4^+^ T cells are seen in patients of advanced age, although it is unclear whether these are a bystander of other sequelae or contribute to the aging process (Hashimoto et al., 2019, Mogilenko et al., 2021). Across infection, autoimmunity, and aging, one common theme is the presence of chronic antigen burden, which may explain the generation or persistence of cytotoxic CD4^+^ T cells in each of these situations, although the contribution of disease-specific contexts must still be carefully considered.

Cytotoxic CD4^+^ T cells are also detected in human cancer, as shown most recently by single-cell genomic surveys, posing important questions as to their biological and clinical relevance. As it is increasingly clear that only a minority of solid tumor patients respond to current immunotherapy approaches such as checkpoint inhibitors, and some tumor types do not respond at all, a deeper understanding of the specific mechanisms and functional regulation of these cytotoxic CD4^+^ T cells may lead to more innovative and effective immunotherapies that are targeted to this effector population independent of or in combination with conventional CD8^+^ T cells. Below, we discuss the current understanding of the ontogeny of cytotoxic CD4^+^ T cells and pre-clinical and clinical evidence for the relevance of these cells in cancer. Based on a comparative review of findings in cancer and non-cancer disease contexts, including single-cell genomic surveys, we synthesize features that define these cells independent of context and highlight key questions specific to human cancer-associated cytotoxic CD4^+^ T cells and their functional programs, regulation, and ontogeny.

## ONTOGENY OF CYTOTOXIC CD4^+^ T CELLS

**Figure 1.**
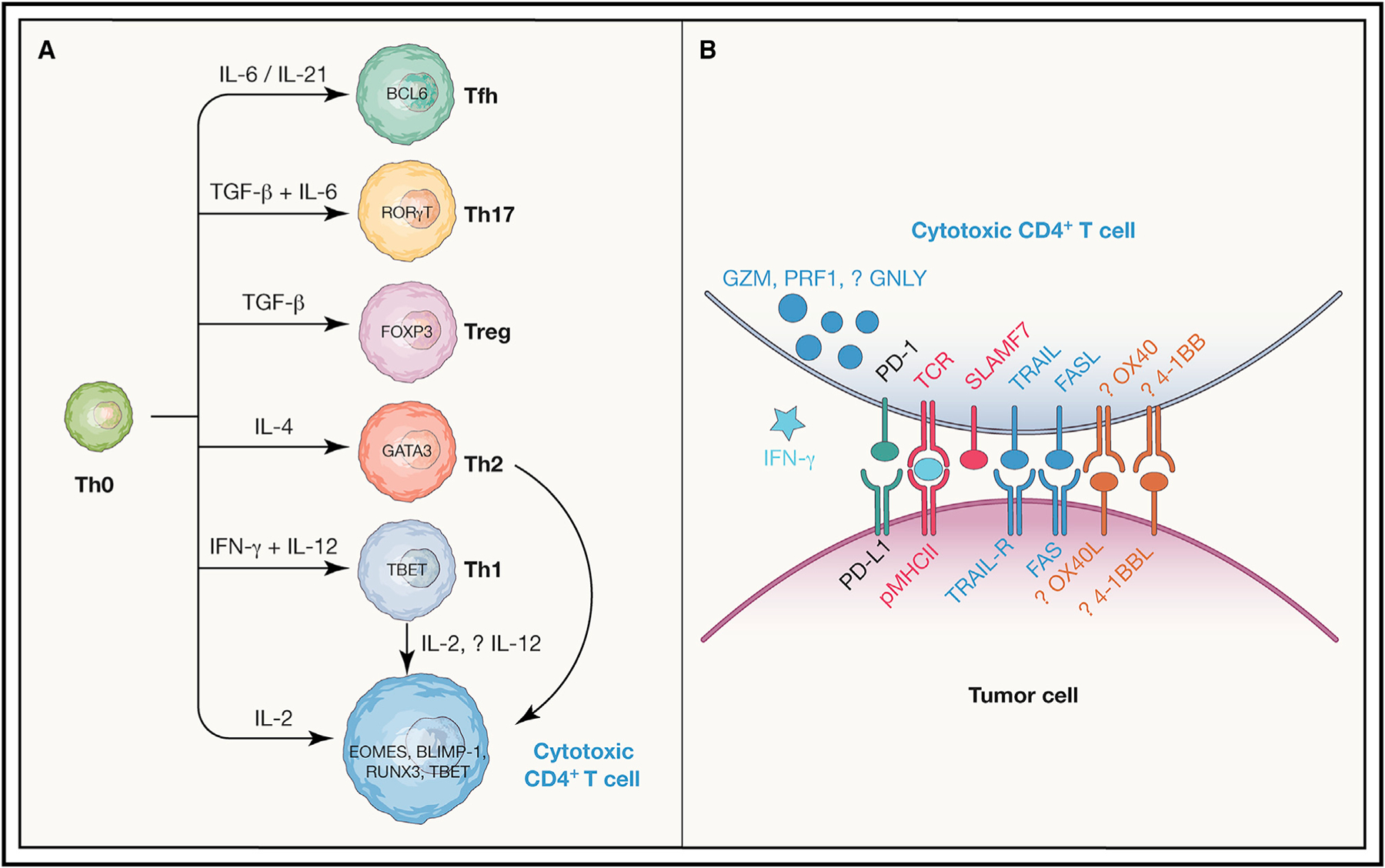
The ontogeny and regulation of cytotoxic CD4^+^ T cells in cancer (A) A schematic of known CD4^+^ T cell functional subtypes, the cytokines that promote their differentiation, and master transcription factors (TFs) involved in their specification. As indicated, cytotoxic CD4^+^ T cells can arise from unpolarized Th0, Th1, or Th2 cells in response to IL-2 depending on the context; also, the contributions of the TFs RUNX3, TBET, BLIMP-1, and EOMES to specifying the cytotoxic CD4+ T cell state are also context-dependent, as discussed in the text. (B) Mechanisms that may contribute to activation of cytotoxic CD4^+^ T cells (red), anti-tumor cytotoxicity effector function (dark blue) including cytokines (light blue), as well as co-stimulatory (orange) or co-inhibitory (green) immune checkpoints are shown.

Most insights into the ontogeny of cytotoxic CD4^+^ T cells have come from studies of anti-viral immunity, models of *in vitro* CD4^+^ T helper (Th) polarization in response to cytokines, and generation of IELs in the intestinal mucosa. Extrinsic factors promote the differentiation of cytotoxic CD4^+^ T cells, notably cytokines (Figure 1A). While cytotoxic CD4^+^ T cells most often exhibit a polyfunctional phenotype associated with co-expression of the Th1 cytokine IFN-γ, this cytokine is not required for the anti-influenza virus response by cytotoxic CD4^+^ T cells (Brown et al., 2012). Moreover, while Th1-polarizing cytokines such as interleukin (IL)-12 can enhance cytotoxic function by increased granzyme and perforin expression in a STAT-4-dependent manner, cytotoxic CD4^+^ T cells can be generated *in vitro* in multiple polarizing conditions (Th0, Th1, and Th2), and in fact, unpolarized Th0 conditions with the addition of IL-2 may be most potent at inducing granzyme B expression and functional cytotoxicity (Brown et al., 2009; Takeuchi et al., 2016). Extrinsic co-stimulation via OX40 is also sufficient to drive the development of cytotoxic CD4+ T cells in murine models, while the addition of 4-1BB co-stimulation aids in the clonal expansion of these cells, including in the context of staphylococcal enterotoxin A (Qui et al., 2011). With regard to CD4^+^ CD8α^+^ IELs that are cytotoxic, intrinsic intestinal cues such as retinoic acid and transforming growth factor (TGF)-β can induce the production of both Treg cells and cytotoxic CD4^+^ T cells (Reis et al., 2013). Class I–restricted T cell–associated molecule (CRTAM), which is also an early activation surface marker for natural killer (NK) and CD8^+^ T cells, identifies a specific subset of CD4^+^ T cells that increase expression of perforin and granzymes upon stimulation, and the cytoplasmic tail of CRTAM is sufficient to enhance the generation of cytolytic CD4^+^ T cells with killing activity post-stimulation (Takeuchi et al., 2016). Finally, the strength of signal transduced through the TCR can itself provide important extrinsic cues, as low antigen dose (when stimulated with IL-2) is optimal for cytotoxic CD4^+^ T cell generation *in vitro* (Brown et al., 2009).

Several transcription factors (TFs) can cooperate to specify the cytotoxic CD4^+^ T cell fate (Figure 1A). The transcription factors BCL6 and TCF7, which specify the development of T follicular helper (Tfh) cells, directly inhibit the development of cytotoxic CD4^+^ T cells in an adenovirus model, suggesting that under these conditions, Tfh and cytotoxic CD4^+^ T cells lie along a common and antagonistic axis (Donnarumma et al., 2016). The TF THPOK specifies CD4^+^ T cell lineage commitment during thymic development, and for cytotoxic CD4+ T cells within IEL, both a loss of the expression of THPOK and coordinate signaling from both TBET (via TBX21) and RUNX3 are required for the generation of these cells (Mucida et al., 2013; Reis et al., 2013). In the context of IL-2-mediated polarization toward cytotoxic CD4^+^ T cells, available evidence suggests that the TF Eomesodermin (EOMES)—which promotes IFN-γ expression and differentiation of CD8^+^ cytotoxic T cells, is directly repressed by THPOK, and is strongly induced by IL-2—is functionally more important to generation of cytotoxic CD4^+^ T cells than the Th1-associated TF TBET. In particular, EOMES appears to be important for the generation of pathogenic cytotoxic CD4^+^ T cells in experimental autoimmune encephalitis (Raveney et al., 2015); also, reduced EOMES expression impairs upregulation of GZMB after *in vitro* activation despite TBET levels being unaffected (Hirschhorn-Cymerman et al., 2012). Finally, in models of cytotoxic CD4^+^ T cell generation enhanced by OX40 and 4-1BB co-stimulation with SEA, EOMES-deficient but not TBX21-deficient T cells are defective in production of GZMB+ CD4^+^ T cells (Qui et al., 2011).

How cytotoxic CD4^+^ T cells differ from conventional CD8^+^ T cells requires further study. Many of the surface receptors and TFs involved in the ontogeny of cytotoxic CD4^+^ T cells are similar to those relevant to CD8^+^ T cell development. However, the function of cytotoxic CD4^+^ T cells may differ from CD8^+^, beyond distinct recognition of MHC class II- versus class I-restricted antigens. For instance, anti-viral cytotoxic CD4^+^ T cells appear to require stimulation to increase and maintain expression of perforin, unlike the constitutive post-thymic expression of this cytolytic molecule in CD8^+^ T cells (Niiya et al., 2005; Pipkin et al., 2010).

The prior body of work characterizing the features, function, and ontogeny of cytotoxic CD4^+^ T cells outside of cancer provides important context but must be carefully interpreted for context dependence when extrapolating to cancer patients. Most insights into the differentiation and functional requirements of cytotoxic CD4^+^ T cells are in the context of viral immunity, which may not involve similar cues to those of cancer; even with the viral context, it is noteworthy that the efficiency of cytotoxic CD4^+^ T cell generation is itself dependent on the specific viral context (e.g., retroviral versus adenoviral; Donnarumma et al., 2016). In addition, whether the findings about TFs instructing the development of CD4^+^ CD8α^+^ IELs are generalizable to other tissue compartments or to cancer requires confirmation.

## CD4+ T CELL RESPONSES IN CANCER IMMUNOTHERAPY—CLINICAL INSIGHTS

Within the cancer context, multiple lines of evidence point to the importance of CD4^+^ T cell recognition of tumor antigens for responses to cancer immunotherapy. Retrospective immune profiling of circulating T cells in patients treated with ipilimumab (anti-CTLA-4) observed an expected pharmacodynamic effect as far as increased absolute lymphocyte counts (ALCs). However, while increased ALC early after the first dose and CD8^+^ T cell percentages later on (between 8 and 14 weeks of treatment) associated with improved survival and clinical response in melanoma patients, increased CD4^+^ T cell percentages at 8–14 weeks also correlated with these positive clinical outcomes (Martens et al., 2016). In a separate trial of metastatic, castration-resistant prostate cancer (mCRPC) patients treated with combinations of ipilimumab plus granulocyte-macrophage colony-stimulating factor (GM-CSF), pharmacodynamic induction of various CD4^+^ and CD8^+^ subsets was also shown, but these changes did not correlate with outcome; instead, pre-existing levels of PD-1+ CD4^+^ T cells in the circulation associated with improved overall survival (Kwek et al., 2015). Moreover, if tumor antigen recognition by CD4^+^ is relevant for outcomes, these tumor antigens should be restricted by MHC class II, and this would predict an association between MHC class II expression and beneficial clinical outcomes. In fact, melanoma-specific MHC class II expression by tumor cells (and not antigen-presenting cells) correlated with both clinical response and overall survival in two separate cohorts of anti-PD-1-treated patients (Johnson et al., 2016). A separate examination of two independent phase II clinical trials of checkpoint inhibition in melanoma similarly found that anti-PD-1 responses associated with increased MHC class II expression and IFN-γ signaling prior to treatment (Rodig et al., 2018).

Another line of evidence for CD4^+^ T cell effector function in anti-cancer responses comes from studies of adoptive cell therapy, in particular chimeric antigen receptor T cell therapy (CAR-T). Specifically, within the realm of currently approved CAR-T therapies for hematologic malignancies, the CD19-directed CAR-T lisocabtagene maraleucel (Bristol Myers Squibb, formerly developed by Juno Therapeutics) is distinct from other approved CAR-T products because the final cell product is designed to have an equal ratio of CD4^+^ and CD8^+^ T cells, both engineered to recognize CD19 in an antigen-independent fashion. While for CD19-directed therapies it remains unclear whether the inclusion of defined proportions of CD4^+^ T cells leads to enhanced efficacy, for a distinct CAR-T targeting B cell maturation antigen (BCMA) on multiple myeloma cells, a post hoc analysis indicated that responses to this therapy were associated with a higher CD4:CD8 ratio in the pre-manufacturing leukapheresis product (Cohen et al., 2019). Although this represents an intriguing observation, the specific phenotype of CD4^+^ subtypes associated with beneficial outcomes is not known, hence, it is unclear whether the productive benefits of CD4^+^ T cells in manufacturing or downstream anti-tumor responses *in vivo* stem from providing help to CD8^+^ T cells, versus direct anti-tumor effector functions.

A more direct indication of the therapeutically beneficial role of CD4^+^ T cells in anti-tumor immunity involves studies of neoantigen vaccination based on whole-exome and RNA-seq to identify candidate neoepitopes generated by non-synonymous somatic mutations (based on predicted MHC affinity), followed by immunization with the top candidates. This generated both CD8^+^ T cell responses to MHC class I-restricted epitopes, but also CD4^+^ T cell responses to MHC class II-restricted epitopes, including in several patients with objective clinical responses or benefit to vaccination (Sahin et al., 2017). Interestingly, in a complementary approach where high-risk resected stage III or stage IV melanoma patients were immunized with pools of long peptides containing predicted neoantigens prioritized based solely on HLA-A or HLA-B predicted binding affinity, neoantigen-specific CD4^+^ T cell responses were seen in addition to CD8^+^ T cell responses, and 10% of immunizing peptides generated both CD4^+^ and CD8^+^ responses (Ott et al., 2017). Furthermore, a pre-clinical study with poorly immunogenic sarcoma lines where either MHC class I- or MHC class II-restricted neoantigens were expressed demonstrated that recognition of MHC class II-dependent neoantigens on tumor cells by CD4^+^ T cells was necessary for tumor rejection in response to checkpoint inhibition and collaborated with MHC class I-dependent neoantigen recognition by CD8^+^ T cells in this context (Alspach et al., 2019).

Several studies involving adoptive cell therapy have also provided direct evidence that CD4^+^ recognition of MHC class II-restricted antigens can provide clinical benefit to cancer patients. One of the earliest reports involved selection and expansion of clonal autologous CD4^+^ T cells from a melanoma patient recognizing an epitope from a shared cancer-testis antigen (NY-ESO-1) presented in the context of HLA-DPB1*0401; infusion of these cells led to a long-term complete response (Hunder et al., 2008). Cell therapy with retrovirally transduced CD4^+^ T cells expressing a TCR specific-for-another-cancer-testis antigen, MAGE-A3, presented by HLA-DPB1*0401 also led to partial and complete response across several tumor types (Lu et al., 2017). Neoantigen-directed CD4^+^ adoptive T cell therapy, where reactive T cells are selected from tumor-infiltrating lymphocytes, also clearly has clinical activity, for instance, in cholangiocarcinoma with a clone directed against a mutation in ERBB2-interacting protein and in urothelial carcinoma with a clone directed against mutated C-terminal binding protein 1 (Tran et al., 2014; Leko et al., 2019). In all of the above reports, transient cytokine-mediated physiology was seen post-infusion, and in several studies, the CD4^+^ T cell clones were confirmed to produce IFN-γ, indicating a Th1-polarized phenotype. It remains unclear whether such CD4^+^ T cell clones are acting by providing help to mobilize other arms of cellular and humoral immunity versus acting directly to kill tumor.

## CYTOTOXIC CD4^+^ T CELLS MEDIATE DIRECT ANTI-TUMOR ACTIVITY IN CANCER

Apart from other roles in providing immunologic help, the importance of the cytotoxic CD4^+^ T cell phenotype in direct tumor killing was first understood in pre-clinical models in which lymphopenic hosts (either RAG-deficient or lethally irradiated) transplanted with syngeneic melanoma tumor received naive, TCR-transgenic CD4^+^ T cells recognizing the TRP-1 antigen. In this context, CD4^+^ T cells produced IFN-γ but also expressed granzymes and perforin and killed tumors in a manner that required GZMB and PRF1, although not Fas ligand or TRAIL, and was enhanced by anti-CTLA-4 (Xie et al., 2010; Quezada et al., 2010). Additionally, in a similar TRP-1 TCR transgenic adoptive transfer model with B16 melanoma in RAG1-deficient lymphopenic hosts, the addition of OX40 agonists and the chemo-therapy cyclophosphamide enhanced tumor killing by cytotoxic CD4^+^ T cells, in a manner dependent on EOMES expression (Hirschhorn-Cymerman et al., 2012). Finally, in both lymphopenic hosts (after lethal irradiation) with the TRP-1 TCR transgenic model and B16 melanoma, the OT-II TCR and B16-OVA melanoma, and immunocompetent hosts implanted with MCA205 sarcoma, the anti-tumor function of GZMB+ CD4^+^ T cells is enhanced by anti-CTLA-4 in a mechanism that involves competition with Treg cells for IL-2 binding (Śledzińska et al., 2020). Notably, the anti-CTLA-4-mediated enhancement of cytotoxic CD4^+^ T cells was independent of TBET (using TBX21-deficient mice) and Th1 functions such as IFN-γ secretion, independent of EOMES (based on lack of detectable protein expression in these cells) but dependent on the alternative transcriptional repressor BLIMP-1 (using conditional PRDM1-deficient mice), which inhibits BCL6 and TCF7 expression in CD4^+^ T cells. Collectively, this work has supported a direct cytotoxic role for CD4^+^ T cells in enhanced anti-tumor immunity after modulation of specific immunity checkpoints (CTLA-4 blockade, OX40 agonism), driven by the effects of TFs that do not involve TBET/Th1 polarization, but may differ depending on the therapeutic context. It is noteworthy that specific pre-clinical models that support a role for CD4-dependent responses to checkpoint inhibition do not invariably support a contribution for CD4-dependent cytotoxicity, as in an immunocompetent model of urothelial carcinoma derived from chemical carcinogenesis where CD4-dependent tumor rejection after combination anti-CTLA-4 plus anti-PD-1 therapy does not require tumor cell expression of MHC class I or II (Sato et al., 2018). This points to a general need for caution when extrapolating to human cancer based on evidence from these pre-clinical models, where the specific biological contribution and mechanisms utilized by cytotoxic CD4^+^ T cells in anti-tumor immunity may depend on the specific tumor type and its associated mutational burden or characteristics as well as the specific immunotherapeutic agent(s) administered and the amount of immunologic space that drives the development of these cytotoxic cells (e.g., lymphopenic versus immunocompetent).

The earliest reports that CD4^+^ T cells could be directly cytotoxic to patient tumor cells involved isolation of circulating CD4^+^ T cell clones specific for NY-ESO-1 in melanoma patients treated with ipilimumab (anti-CTLA-4). Several of these clones were first identified for the ability of OX40 engagement to enhance their *in vitro* killing of autologous melanoma tumor cells (Hirschhorn-Cymerman et al., 2012). In a subsequent report, several cytotoxic CD4^+^ clones were identified based on selection for Th1 phenotypes by ELISPOT; two patients who were seropositive for responses to NY-ESO-1 at baseline also had NY-ESO-1-reactive pre-treatment CD4^+^ T cell clones, while two other patients did not have pre-treatment humoral or cellular CD4^+^ responses to NY-ESO-1, but all responses were enhanced by ipilimumab therapy. CD4^+^ T cell lines established from all four patients from peripheral blood mononuclear cells (PBMCs) after ipilimumab therapy expressed granzyme B, perforin, and LAMP (CD107a) and were able to lyse autologous melanoma cells in a manner dependent on MHC class II recognition; analysis of lines established from longitudinal samples before or after ipilimumab from one patient demonstrated that the induction of cytolytic granzyme and perforin occurred only after ipilimumab therapy (Kitano et al., 2013).

Focusing on evidence for the contribution of cytotoxic CD4^+^ T cells in human cancer, recent unbiased studies of the T cell compartment in cancer patients, using scRNA-seq, have documented cytotoxic CD4^+^ T cells that express cytolytic effector molecules such as granzymes (*GZMA*, *GZMB*, *GZMH*, *GZMK*), perforin (*PRF1*), and other granule-associated proteins such as *NKG7* and granulysin (*GNLY*), both in the tumor and in the circulation of patients with multiple solid tumors (non-small-cell lung cancer, colorectal cancer, hepatocellular carcinoma, bladder cancer, osteosarcoma, breast cancer, and head and neck cancer) (Azizi et al., 2018; Guo et al., 2018; Oh et al., 2020; Puram et al., 2017; Zhang et al., 2018, 2019, 2021; Zhou et al., 2020; Table 1).

scRNA-seq of tumor-infiltrating lymphocytes (TIL) reveals discrete states of cytotoxic CD4^+^ T cells expressing cytolytic proteins in human bladder cancer. While all populations expressed *GZMA* and other granule-associated proteins such as *GNLY* and *NKG7*, distinct states co-expressed either *GZMB* or *GZMK*, and *PRF1* was more predominantly co-expressed with *GZMB*. These findings were confirmed by multiplexed immunofluorescence staining of a subset of the same tumors and flow cytometry of a separate set of tumors. Notably, cytotoxic CD4^+^ T cells in bladder tumors were also polyfunctional, with over half of cells able to express both IFN-γ and tumor necrosis factor (TNF)-α with *ex vivo* stimulation. These cytotoxic CD4^+^ T cell subsets exhibited a clonally expanded repertoire in tumor compared with paired adjacent normal bladder tissue. This suggested an effector role for this population in response to specific antigens. Moreover, these cells directly killed autologous bladder tumors in a manner dependent on MHC class II recognition, with kinetics similar to autologous CD8^+^ killing. Expansion of these cytotoxic CD4^+^ T cells in the presence of autologous Treg cells resulted in diminished cytotoxic activity, establishing the cytotoxic CD4-to-Treg ratio as another relevant axis of intra-tumoral immune activity. Indeed, the presence of cytotoxic CD4^+^ T cells in pre-treatment tumors, based on expression of a gene signature associated with these cells, was predictive of clinical response to anti-PD-L1 in bladder cancer (Oh et al., 2020).

The capacity of cytotoxic CD4^+^ T cells to kill tumor has also been demonstrated in melanoma patients. Single-cell cytotoxicity assays of cytotoxic CD4^+^ T cells specific for tumor antigen (NY-ESO-1 and MAGE-A3), which were isolated using MHC class II tetramers, demonstrated their direct killing of tumor cells pre-treated with IFN-γ or stably transduced with CIITA to enhance MHC class II expression (Cachot et al., 2021). This killing was directly dependent on granzyme activity, and incubation with an agonistic antibody directed against SLAMF7 partially enhanced cytotoxicity. This molecule was also identified in the predictive cytotoxic CD4^+^ T cell-associated signature in bladder cancer (Oh et al., 2020). Finally, in comparative quantitative PCR analysis of cytotoxic versus helper CD4^+^ T cell clones, *RUNX3* was significantly enriched in cytotoxic cells, while other TFs such as *THPOK*, TBET (ie *TBX21*), *GATA3*, *EOMES*, and BLIMP-1 (i.e., *PRDM1*) were not (Cachot et al., 2021).

This evidence for direct CD4^+^ T cell cytotoxicity against patient tumors does not preclude other CD4-dependent effector functions in anti-tumor immunity. CD4^+^ T cells can differentiate into multiple effector states in addition to the cytotoxic phenotype that can impact immunotherapy responses in cancer (Figure 1A). Th1-polarized ICOS+ CD4^+^ T cells that produce IFN-γ further infiltrate urothelial tumors in ipilimumab-treated patients (Liakou et al., 2008). Th17 CD4^+^ cells can either promote tumorigenesis or protect against established tumors depending on the context (reviewed in Vitiello and Miller, 2020), although several lines of evidence indicate that Th17 CD4^+^ T cells can eradicate tumors including when modified as CAR-T cells (Nelson et al., 2020; Guedan et al., 2014; reviewed in Knochelmann et al., 2018). Regarding the T follicular helper (Tfh) phenotype, while inhibitory PD-1+ CD4^+^ T cells resembling Tfh cells are blocked by anti-PD-1 therapy (Zappasodi et al., 2018), there is evidence to the contrary that Tfh cells can mediate response to checkpoint inhibition in genetically engineered mouse models (Hollern et al., 2019). Finally, there is clear evidence that productive cancer immunotherapy can impact the frequencies and function of Treg cells to promote effective anti-cancer immunity. However, taken together, recent single-cell genomic and functional studies in patients have supported a direct role for CD4^+^ T cells in killing of diverse patient tumor types that is dependent on MHC class II recognition and granzymes and has provided some important initial insights into activating co-receptors (SLAMF7) or inhibitory inputs (e.g., by autologous Tregs) that regulate their activity.

## COMPARATIVE FEATURES OF CYTOTOXIC CD4^+^ T CELL EFFECTOR CELLS IN HUMAN CANCER AND NON-CANCER CONTEXTS

**Figure 2.**
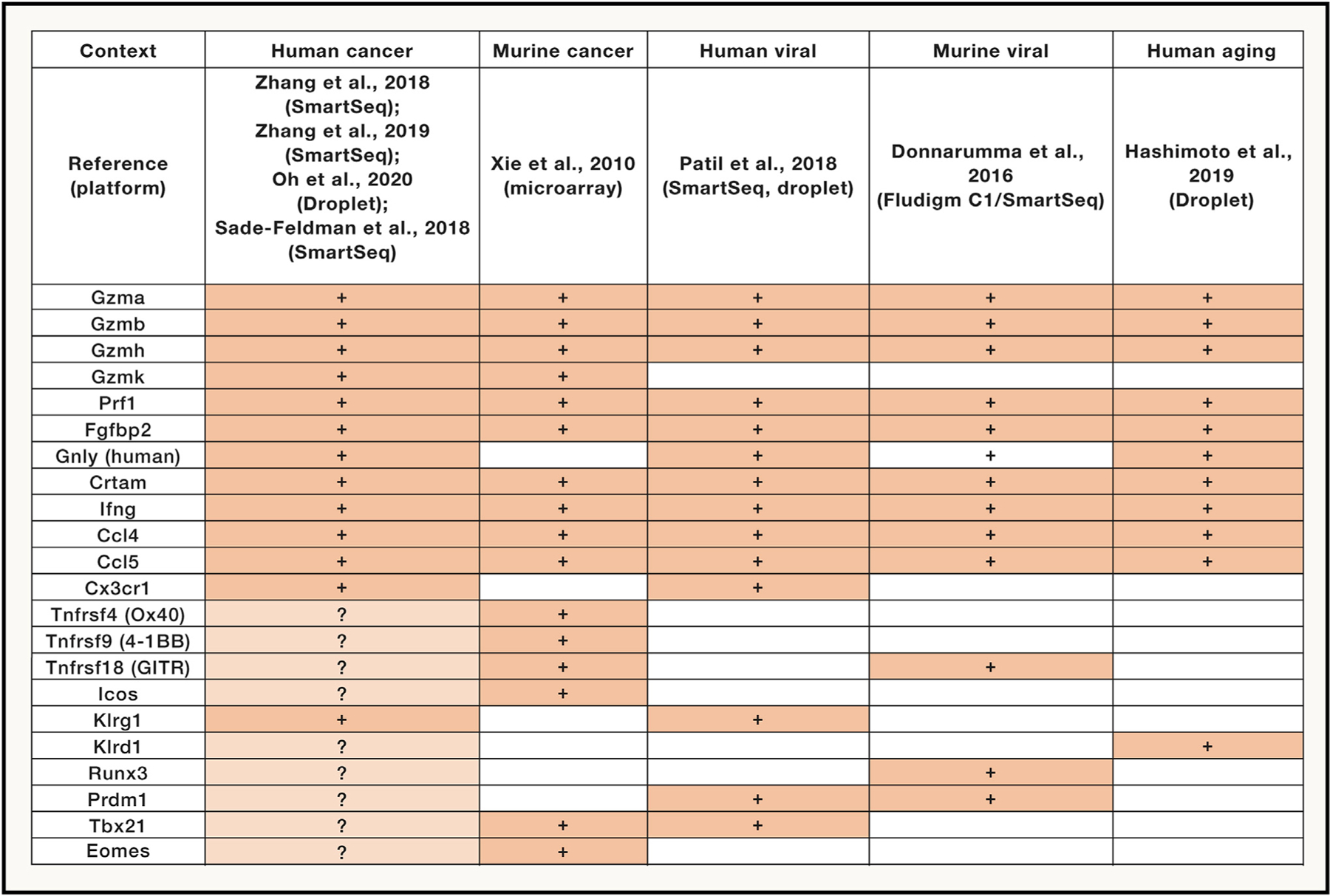
Comparison of cytotoxic CD4^+^ T cell programs in cancer and non-cancer contexts Expression of key transcripts in cytotoxic CD4^+^ T cells in human and murine cancer, as well as human viral (dengue), murine viral (adenovirus), and human aging. Comparisons were made based on inspection of single-cell transcriptomic data, with the exception of bulk microarray data for murine cancer. Specific references for each dataset, and the platforms used for single-cell sequencing, are indicated. Lighter shading and question marks indicate where findings are inconsistent between datasets (e.g., comparing various human cancers).

As single-cell transcriptomic data are increasingly available, a comparison from a subset of representative studies is worth-while to obtain some holistic insights into mechanisms and pathways that are shared or distinct in cytotoxic CD4^+^ T cells in human cancer versus non-cancer or murine cancer contexts. However, comparative analysis across these contexts is limited by significant technical confounders, specifically different sequencing platforms with superficial (droplet-based) or deep (SmartSeq) sequencing depth, the use of distinct comparator populations to determine the genes differentially expressed in cytotoxic CD4^+^ T cells, as well as different cell and sample numbers that affect statistical power to determine genes enriched in these cells. In addition, the comparison between cancer and non-cancer contexts is inherently limited by biological differences in circulating versus tumor-infiltrating populations (i.e., all T cell phenotypic data from non-cancer is largely from circulating cells). These are important caveats in interpreting these comparisons, as these limitations directly impact one’s confidence in saying that a specific marker gene positively expressed in one context is definitively absent in the other context (versus simply unable to be detected). Nonetheless, a number of shared features, and some possibly divergent programs, can be seen in cross-context comparisons of cytotoxic CD4^+^ T cells in cancer and non-cancer contexts (Figure 2).

The core module of cytolytic effector molecules appears largely conserved across murine and human viral, human aging, and human cancer contexts: notably, *GZMB*+ cytotoxic CD4+ T cells co-express *GZMA* and often *GZMH*, *PRF1*, *FGFBP2*, and granule-associated proteins *NKG7* and (in humans) *GNLY* (Donnarumma et al., 2016; Patil et al., 2018; Hashimoto et al., 2019; Zhang et al., 2018, 2019; Oh et al., 2020; Cachot et al., 2021). Notably, with increased clustering resolution, a separate population of *GZMK*+ CD4^+^ T cells can be found in human TIL (bladder, colorectal), where they express a distinct complement of effector molecules, specifically, high levels of *CRTAM* and relatively lower levels of *PRF1* (Zhang et al., 2018; Oh et al., 2020). Both *GZMB*+ and *GZMK*+ CD4^+^ subsets appear to co-express *IFNG* consistent with polyfunctionality (Zhang et al., 2018; Oh et al., 2020). These findings are roughly consistent with the microarray phenotype of TRP-1 transgenic cytotoxic CD4^+^ T cells obtained from lymphopenic mice with regressing melanoma tumors (Xie et al., 2010), although comparisons are limited between this prior bulk microarray data and single-cell transcriptomic data.

Another conserved feature of cytotoxic CD4^+^ T cells across these cancer and non-cancer contexts is the co-expression of chemokines, usually *CCL4* and *CCL5*, although the biological importance of this production is unknown; in some studies, expression of the chemokine receptor *CX3CR1* (fractalkine receptor) is characteristic of *GZMB*+ CD4^+^ T cells in both dengue viral infection and colorectal cancer (Patil et al., 2018; Zhang et al., 2018).

As far as immune checkpoint expression, the general consensus is that cytotoxic CD4^+^ T cells are lacking in expression of many conventional checkpoints at the level of transcript at least in human cancer (Zhang et al., 2018, 2019; Oh et al., 2020), although individual studies from human melanoma TIL point to some degree of expression of *TNFRSF4* (OX40/CD134), *TNFRSF9* (4-1BB/CD137), and *TNFRSF18* (GITR) (Cachot et al., 2021, based on re-clustering of data from Sade-Feldman et al., 2018). Some level of GITR expression may be a common feature between human cancers and murine models of adenovirus where cytotoxic CD4^+^ T cells are induced (Donnarumma et al., 2016). As far as other co-stimulation, while *ICOS* expression has been seen in human melanoma TIL for cytotoxic CD4^+^ T cells (Cachot et al., 2021), this is not universally seen across the other cancer/non-cancer studies.

Killer-type lectins are also of interest as markers of cytotoxic CD4^+^ T cells, and in the *GZMB*+ subset, *KLRG1* is consistently associated with these cells in both human cancers (Zhang et al., 2018; Cachot et al., 2021) and human dengue viral infection (Patil et al., 2018), consistent with an effector phenotype. In some cases, *KLRD1* can also been seen in human cancer on GZMB+ CD4^+^ T cells (Oh et al., 2020), which is also seen in aging humans (Hashimoto et al., 2019).

Finally, the pattern of master TF expression in cytotoxic CD4^+^ T cells is variable across these contexts, which may in part be related to sensitivity of detection with droplet-based versus deep sequencing. For a mixed population of *GZMB*+ and *GZMK*+ CD4^+^ in melanoma, higher *RUNX3* and *PRDM1* (BLIMP-1) expression was predominant over low-level *TBX21*/*EOMES* expression (Cachot et al., 2021), and the *PRDM1* overexpression is supported by similar findings from *GZMB*+ CD4^+^ T cells in murine adenoviral infection (Donnarumma et al., 2016). However, other studies have found the reverse with *TBX21*-predominant expression specifically in *GZMB*+ CD4^+^ T cells in both human colorectal cancer and dengue viral infection (Zhang et al., 2018; Patil et al., 2018). In regard to TFs in *GZMK*+ CD4^+^ T cells, our understanding is incomplete; while one report identified overexpression of both *EOMES* and *RUNX3* in human colorectal cancer, generally, these and other TFs were not detected in other cancer contexts (Oh et al., 2020), and in the human dengue viral context, *PRDM1* was more associated with the circulating *GZMK*+ CD4^+^ T cell subset (Patil et al., 2018). Overall, while there are clearly conserved features of cytotoxic CD4^+^ T cells across both cancer and other immune contexts, and some possible divergent features, this requires further study to better elucidate the specific program and activation requirements in the context of human cancer.

## ACTIVATION AND FUNCTION OF CYTOTOXIC CD4^+^ T CELLS IN HUMAN CANCER

A number of important outstanding questions remain regarding the biology and regulation of cytotoxic CD4^+^ T cells in cancer patients. Regarding the fundamental question of how cytotoxic CD4^+^ T cells kill human tumor target cells, the prior data from cytotoxic CD4^+^ T cells in murine models points to contact-dependent cytotoxicity in a granzyme- and MHC class II-dependent fashion, where the action of IFN-γ is dispensable (Figure 1B). In patients, although MHC class II-dependent antigen recognition is a consistent finding, it is worth noting that in some studies, the degree of impact on killing with MHC class II-blocking antibodies is partial (Oh et al., 2020), and that in other studies, functional studies were performed with target cells where MHC class II expression was enhanced or stabilized (Cachot et al., 2021), leaving open the possibility of some degree of bystander tumor killing in a non-MHC-restricted manner, which could be relevant in tumor cells lacking MHC class II. As far as the efficacy of MHC class II-dependent killing, of note, although the repertoire of naturally occurring cytotoxic CD4^+^ T cells in bladder cancer is noted to be restricted compared with other CD4^+^ T cell subsets (Oh et al., 2020), even within multimer-selected cytotoxic CD4^+^ T cell lines, the repertoire is polyclonal (Cachot et al., 2021), and it is likely that only a select number of TCR clonotypes are of sufficient avidity to trigger killing. As far as effector molecules used by these cells to trigger death, a consistent finding is that these cytotoxic CD4^+^ T cells are polyfunctional and express IFN-γ and TNF-α in addition to cytolytic effector proteins. While the most recent work from Cachot et al. characterizing cytotoxic CD4^+^ T cell clones identified necessary roles for perforin and granzyme B in MHC class II-dependent target killing (based on pharmacologic inhibition with concanamycin A and compound 20), it is worth noting that this inhibition was partial, and that compared with conventional helper CD4^+^ T cell clones, the cytotoxic clones also had higher maximal levels of production of IFN-γ and TNF-α. Furthermore, while expression of surface death receptors such as Fas ligand or TRAIL was not correlated with higher cytotoxic potential, other pathways (type I cytokines, death ligands) may make a tonic contribution to target cell death in collaboration with contact-dependent granule exocytosis. It is possible that in addition to GZMB and PRF1, other granule constituent proteins may contribute to death (Figure 1B), although this has not been directly tested. Cytotoxic CD4^+^ T cells also express GNLY, which has a documented role in anti-microbial immunity (reviewed in Krensky and Clayberger, 2009); although a proven role in anti-tumor responses has not been documented, profiling of single-cell clones with cytotoxic activity pointed to an enrichment of *GNLY* expression in this population (Cachot et al., 2021). Additional granule constituents include the protein NKG7 (Medley et al., 1996) that is commonly seen in cytotoxic effector cells, including cytotoxic CD4^+^ T cells, whose function has been elusive.

In addition to cognate antigen recognition by TCR, it is likely that other co-receptors collaborate to determine the activation setpoint for these cells, and that while there may be conserved mechanisms, tumor-specific mechanisms will likely come into play (Figure 1B). While evidence points to SLAMF7 as an activating co-receptor in melanoma (Cachot et al., 2021), and part of a cytotoxic CD4^+^ T cell-associated gene signature associated with checkpoint inhibitor response (Oh et al., 2020), CD4^+^ T cell expression of this receptor based on scRNA-seq was absent in some tumors (Cachot et al., 2021), and this will need to be verified at the level of protein expression in individual tumor contexts. Also of interest are inhibitory receptors that may be engaged by tumor- or tumor stromal-associated ligands, and therefore act as additional “checkpoints” to inhibit cytotoxic CD4^+^ T cell activity. The prototypical immune checkpoint PD-1 is variably expressed at the transcript or protein level in human cytotoxic CD4^+^ T cells from cancer patients (Kitano et al., 2013; Oh et al., 2020; Cachot et al., 2021), and its surface expression decreases in longitudinal cytotoxic CD4^+^ T cell clones isolated from individual patients following ipilimumab treatment in concert with enhanced cytotoxic activity (Kitano et al., 2013). Together with the predictive value of a cytotoxic CD4^+^ T cell signature for anti-PD-L1 response in bladder cancer (Oh et al., 2020), this suggests a role for PD-1 blockade in enhancing the activity of these cells. However, as noted above, many other conventional immune checkpoints such as *TIGIT*, *TNFRSF4* (OX40 receptor/CD134), *TNFRSF9* (4-1BB/CD137), and *TNFRSF18* (GITR) are not universally overexpressed in cytotoxic CD4^+^ T cells from cancer patients (Oh et al., 2020). Hence, depending on the disease context, the co-stimulation-induced enhancement of cytotoxic CD4^+^ T cells seen in murine models may not apply, which points to the need to understand their distinct activation requirements in patients for purposes of therapeutic enhancement. Additionally, transcriptomics will not fully reflect surface protein expression of co-receptors or ligands on cytotoxic CD4^+^ T cells and tumors, respectively, and will require candidate or unbiased proteomic approaches integrating model organisms and *ex vivo* patient samples for discovery, followed by functional validation in suitable *ex vivo* or *in vivo* model systems.

A key unanswered question is the relative contribution of cytotoxic CD4^+^ T cells *in vivo* for either immunosurveillance against tumor progression, or response to immunotherapy, set against the functional contributions of other immune effector populations. Earlier pre-clinical work studying expansion of cytotoxic CD4^+^ T cell in lymphopenic hosts may point to situations where, if CD8-mediated immunity is impaired, cytotoxic CD4^+^ T cell phenotypes can expand to provide protection, although this has not been studied directly in patients (Xie et al., 2010; Quezada et al., 2010; Hirschhorn-Cymerman et al., 2012). The specific contribution of cytotoxic CD4^+^ T cells in direct anti-tumor cytotoxicity in an immune-competent scenario with the presence of other cytotoxic effectors remains to be determined. Within the infectious disease literature, there are specific instances in which memory CD4^+^ T cells that can kill virus-infected cells in a perforin-dependent manner when other lymphocytes are absent and can synergize with naive CD8^+^ to enhance clearance of influenza in a manner that does not require expression of IFN-γ (McKinstry et al., 2012). Similar dissection of cytotoxic CD4^+^ T cell-dependent anti-tumor activity and how this may synergize with other anti-tumor effectors will be illustrative.

## ONTOGENY OF CYTOTOXIC CD4^+^ T CELLS IN HUMAN CANCER AND ITS RELATIONSHIP TO CD8^+^ T CELLS

Data from either *in vitro* IL-2 differentiation or murine models of cancer seem to suggest that while TBET (and associated Th1 functions such as IFN-γ secretion) may be dispensable for cytotoxic CD4^+^ T cell generation and function, whether EOMES or BLIMP-1 is important may depend on the specific conditions and disease context; while it is noted that TBET and RUNX3 are critical for generation of cytotoxic CD4^+^ IELs, it is unclear how this may generalize to cytotoxic CD4^+^ T cells in human cancer. Meanwhile, *ex vivo* studies of cytotoxic CD4^+^ T cells from human cancer patients points to variable overexpression of either *TBX21* or *RUNX3* plus BLIMP-1 (via *PRDM1*) when compared with non-cytotoxic CD4^+^ T cells. RUNX3 is intriguing as it is involved in specifying the conventional cytotoxic CD8+ T cell lineage upstream of EOMES (Cruz-Guilloty et al., 2009), but future directions will require direct perturbation of these axes *ex vivo* to determine the relative contribution of these TFs in specifying the cytotoxic CD4^+^ T cell fate. It is noteworthy that developmental relationships can be inferred from analysis of scRNA-seq data with paired single-cell TCR data to track discrete single-cell functional states linked by the same clonotype; this points to marked plasticity of cytotoxic CD4^+^ T cells, which can share TCR specificity with distinct effector memory or proliferating CD4^+^ T cells (Zhang et al., 2018; Oh et al., 2020). This suggests that the generation of cytotoxic CD4^+^ T cells may be a complex and ongoing dynamic process with distinct microenvironmental cues in cancer patients.

## CONCLUDING REMARKS

The recognition that CD4^+^ T cells can also mediate cytotoxicity in cancer should lead to novel approaches to further enhance their direct anti-tumor activity in patients. However, these strategies must be critically guided by a deeper understanding of the biology of cytotoxic CD4^+^ T cells in human cancer. While our comparative analysis of cytotoxic CD4^+^ T cells across cancer and non-cancer contexts reveals some conserved effector cytolytic functions (and MHC class II-dependent recognition), as well as overlap in master TFs associated with this CD4^+^ T cell state, the available evidence also highlights at least two distinct challenges for their therapeutic targeting. First, across contexts, these cells generally lack expression of conventional immune checkpoints, which are current targets for intervention via checkpoint inhibitors. In addition, the data from cancer patients points to divergent mechanisms for their ontogeny, regulation, and cytolytic effector function that may differ from other non-cancer contexts or murine models and may in fact be highly dependent on the specific immune context of individual cancer subtypes. Moreover, many tumor cells lack MHC class II expression, so the effects of cytotoxic CD4^+^ T cells may be less relevant for these tumors or may rely on bystander killing on antigen-presenting cells, which could also lead to immune modulation with the tumor. These prompt a number of key outstanding questions (see Box 1) regarding the biology of cytotoxic CD4^+^ T cells in human cancer that must be carefully weighed in future correlative or *ex vivo* functional studies of these cells. A deeper understanding of this biology may lend itself to numerous therapeutic avenues including whether *ex vivo* enhancement of their cytolytic effector functions based on a deeper knowledge of the underlying signals, co-administration as adoptive cell therapy with agents targeting cytotoxic CD4^+^ T cell-specific surface receptors to enhance their activation or block inhibitory surface receptors, identification of specific signaling nodes that may block Treg-specific inhibition of cytotoxic CD4^+^ T cell function *in vivo*, or development of therapies that can enhance cytotoxic CD4^+^ T cell-specific effector functions in the context of other immunotherapy approaches (CAR-T therapies, neoantigen-targeted vaccines, or cell therapies).

## Figures and Tables

**Table 1.** Cytotoxic CD4^+^ T cells in human cancers

Cancer type	Reference
Non-small-cell lung cancer	Guo et al., 2018
Colorectal cancer	Zhang et al., 2018
Hepatocellular carcinoma	Zhang et al., 2019
Bladder cancer	Oh et al., 2020
Melanoma	Hirschhorn-Cymerman et al., 2012; Kitano et al., 2013; Cachot et al., 2021; Sade-Feldman et al., 2018
Breast cancer (various)	Azizi et al., 2018; Zhang et al., 2021
Osteosarcoma	Zhou et al., 2020
Head and neck cancer	Puram et al., 2017

## Box 1. Key outstanding questions regarding cytotoxic CD4^+^ T cells in human cancer

- How does the phenotype of cytotoxic CD4^+^ T cell effectors in human cancer compare with either murine models of cancer or non-cancer contexts?
- How do cytotoxic CD4^+^ T cells kill tumor cells?
- What are other novel mechanisms by which cytotoxic CD4^+^ T cells can contribute to killing of tumor cells?
- How is the activation of cytotoxic CD4^+^ T cells regulated in human cancer?
- What is the relevant biological context in which cytotoxic CD4^+^ can provide protection against tumor progression or regression in response to immunotherapy?
- What is the ontogeny of cytotoxic CD4^+^ T cells relative to their CD8^+^ counterparts in cancer patients?
